# Phylogenetics links monster larva to deep-sea shrimp

**DOI:** 10.1002/ece3.347

**Published:** 2012-08-24

**Authors:** Heather D Bracken-Grissom, Darryl L Felder, Nicole L Vollmer, Joel W Martin, Keith A Crandall

**Affiliations:** 1 Department of Biology, Brigham Young UniversityProvo, Utah, 84602; 2 Department of Biology, Florida International University-Biscayne Bay CampusNorth Miami, Florida, 33181; 3 Department of Biology, University of Louisiana at LafayetteLouisiana, 70504; 4 Southeast Fisheries Science Center, National Marine Fisheries Service, NOAALafayette, Louisiana, 70506; 5 Natural History Museum of Los Angeles CountyLos Angeles, California, 90007; 6 Computational Biology Institute, George Washington UniversityAshburn, Virginia, 20147

**Keywords:** *Cerataspis monstrosa*, Decapoda, DNA barcoding, larval–adult linkage, phylogenetics

## Abstract

Mid-water plankton collections commonly include bizarre and mysterious developmental stages that differ conspicuously from their adult counterparts in morphology and habitat. Unaware of the existence of planktonic larval stages, early zoologists often misidentified these unique morphologies as independent adult lineages. Many such mistakes have since been corrected by collecting larvae, raising them in the lab, and identifying the adult forms. However, challenges arise when the larva is remarkably rare in nature and relatively inaccessible due to its changing habitats over the course of ontogeny. The mid-water marine species *Cerataspis monstrosa* (Gray 1828) is an armored crustacean larva whose adult identity has remained a mystery for over 180 years. Our phylogenetic analyses, based in part on recent collections from the Gulf of Mexico, provide definitive evidence that the rare, yet broadly distributed larva, *C. monstrosa*, is an early developmental stage of the globally distributed deepwater aristeid shrimp, *Plesiopenaeus armatus*. Divergence estimates and phylogenetic relationships across five genes confirm the larva and adult are the same species. Our work demonstrates the diagnostic power of molecular systematics in instances where larval rearing seldom succeeds and morphology and habitat are not indicative of identity. Larval–adult linkages not only aid in our understanding of biodiversity, they provide insights into the life history, distribution, and ecology of an organism.

## Introduction

**Figure 1:**
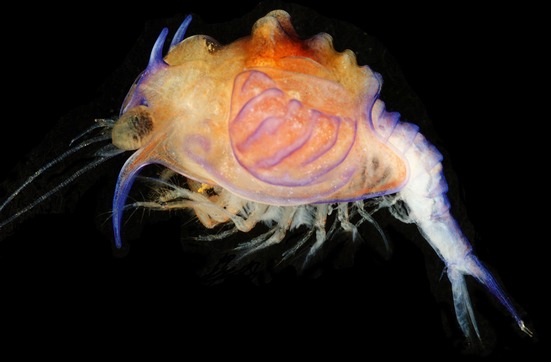
*Cerataspis monstrosa* (median carapace length 11.8 mm), the ‘monster’ larva that has remained unlinked to an adult form for 184 years. The photographed and analyzed specimen was collected on July 2 2009 in the northeastern Gulf of Mexico over a depth of 420 m at 27˚05.996′N, 86˚25.916′W during mid-water trawl collection by cruise participants aboard NOAA Ship Gordon Gunter. [Credit: D. Felder, 2011]

Exploration of our largely unknown oceans continues to yield fascinating biodiversity discoveries. In addition to novel forms of life (Osborn et al. 2009), chance collecting coupled with modern molecular genetic tools allow us to better understand longstanding enigmas. For over 180 years, the “monster” larva, *C. monstrosa*, has been such a puzzle to zoologists. This species, first discovered in the gut contents of a dolphin in 1828 (Gray 1828), is unique in its heavy armor, thick body, and exceptional horn ornamentation (Fig. 1). Nineteenth century collections of marine plankton commonly included developmental stages of crabs, shrimps, and lobsters that differed strikingly from their adult counterparts in morphology and habitat (Williamson 1915; Gurney 1939,1942; Anger 2001). Not originally identified as a larval decapod, the single specimen of *C. monstrosa* was described as a “monstrous and misshapen animal” and placed within a new genus and species of primitive crustacean (Leptostraca) (Gray 1828). Although many such larvae have been subsequently linked to adult forms, *C. monstrosa* has eluded definitive placement despite nearly two centuries of effort due to its scarcity and extreme morphological uniqueness.

*Cerataspis monstrosa* is encountered only rarely in the wild with most information on this species coming from studies of gut contents of its predators, including skipjack (*Katsuonus pelamis*), yellowfin (*Thunnus albacares*) and blackfin (*T. atlanticus*) tuna, and dolphin (*Coryphaena hippurus*) (Morgan et al. 1985). Interpretations of its unusual morphology have to date suggested it might represent the larval counterpart of some abyssal adult, the proposed candidates being a yet-to-be discovered shrimp from the family Aristeidae (Penaeoidea), or perhaps even a more distant relative of penaeoids (Heegaard 1966a; Osborn et al. 2009; Hubert et al. 2010). Wild-caught planktonic larvae are often collected and reared to early postlarval stages in order to determine their adult identities (Gurney 1942; Rice and Williamson 1970). However, in the case of deep oceanic species, with highly metamorphic development involving striking vertical migrations between near-surface and deep-ocean waters, rearing protocols seldom succeed. In these instances, DNA data provide a common currency for comparison (Webb et al. 2006; Ahrens et al. 2007; Burns et al. 2008; Hubert et al. 2010).

Recently, mid-water oceanic collections in the northern Gulf of Mexico unexpectedly included a single specimen of *C. monstrosa* suitable for genetic analyses. We collected DNA sequence data from this specimen to compare to data in our extensive database of decapod crustacean DNA sequences (http://decapoda.nhm.org/, Table 1). Taxon selection was based on previous studies that suggested a relationship between *Cerataspis* and shrimp-like decapods. By the late nineteenth century, an affinity between *Cerataspis* to penaeoid shrimp had been proposed (Dohrn 1871; Giard and Bonnier 1892; Heegaard 1966b), and by the early twentieth century, new observations suggested this peculiar form represented a protracted pelagic larval stage of the family Aristeidae (Bouvier #b^101^). As previous studies suggested an affinity between *Cerataspis* and penaeoid shrimp, and more specifically the family Aristeidae, we sampled heavily within these groups (Boas 1880; Giard and Bonnier 1892; Bouvier #b^101^; Burkenroad 1934).

**Table 1:** Taxonomy, voucher catalog numbers, and GenBank (GB) accession numbers for gene sequences used in the study. An “N/A” (not available) indicates missing sequence data. New sequences are indicated in bold

Taxon		GB nos.				
		
	Voucher	12S	16S	18S	28S	H3
Outgroup taxa						
Euphausiacea Dana, 1852						
Euphausiidae Dana, 1852						
*Euphausia* sp.	ULLZ8093	N/A	EU868655	EU868746	**JX403819**	**JX403899**
Stenopodidea Claus, 1872						
Stenopodidae Claus, 1872						
*Stenopus hispidus* (Olivier, 1811)	KC4276	**JX403879**	**JX403856**	FJ943443	FJ943450	FJ943457
Caridea Dana, 1852						
Procarididae Chace & Manning, 1972						
*Procaris ascensionis*						
Chace & Manning 1972	KC4274	**JX403877**	GQ487495	GQ487503	GQ487511	GQ487521
Atyidae de Haan, 1849						
*Atyopsis* sp.	ULLZ9174	**JX403874**	EU868634	EU868724	**JX403817**	**JX403897**
Hippolytidae Dana, 1852						
*Latreutes fucorum* (Fabricius, 1798)	ULLZ9135	**JX403873**	EU868664	EU868755	**JX403816**	**JX403896**
Ogyrididae Holthuis, 1955						
*Ogyrides* nr. *alphaerostris*	ULLZ7755	**JX403875**	EU868679	EU868772	**JX403818**	**JX403898**
Ingroup taxa						
Penaeoidea Rafinesque-Schmaltz, 1815						
Aristeidae Wood-Mason, 1891						
*Aristaeomorpha foliacea* (Risso, 1827)	KC4280	**JX403863**	GQ487491	GQ487500	GQ487508	GQ487517
*Aristaeopsis edwardsiana* (Johnson, 1868)	ULLZ7726	**JX403872**	**JX403854**	**JX403836**	**JX403815**	**JX403895**
*Cerataspis monstrosa* Gray, 1828	ULLZ11555	**JX403884**	**JX403860**	**JX403842**	**JX403824**	**JX403904**
*Hemipenaeus carpenteri* Wood-Mason, 1891	ULLZ8551	**JX403865**	**JX403847**	**JX403829**	**JX403808**	**JX403889**
*Plesiopenaeus armatus* (Bate, 1881)	ULLZ11940	**JX403876**	**JX403855**	**JX403837**	**JX403820**	**JX403900**
Benthesicymidae Wood-Mason, 1891						
*Bentheogennema intermedia* (Bate, 1888)	ULLZ6701	**JX403869**	**JX403851**	**JX403833**	**JX403812**	**JX403892**
*Benthesicymus bartletti* Smith, 1882	ULLZ8036	**JX403887**	N/A	**JX403845**	**JX403827**	N/A
*Gennadas valens* (Smith, 1884)	ULLZ11476	**JX403882**	**JX403858**	**JX403840**	**JX403822**	**JX403902**
Penaeidae Rafinesque, 1815						
*Farfantepenaeus duorarum* (Burkenroad, 1939)	ULLZ8365	**JX403864**	**JX403846**	**JX403828**	**JX403807**	**JX403888**
*Funchalia villosa* (Bouvier, 1905)	ULLZ6700	**JX403870**	**JX403852**	**JX403834**	**JX403813**	**JX403893**
*Litopenaeus setiferus* (Linnaeus, 1767)	ULLZ11629	**JX403886**	**JX403862**	**JX403844**	**JX403826**	**JX403906**
*Litopenaeus vannamei* (Boone, 1931)	KCpen	EU920908	EU920934	EU920969	EU921005/EU921006	EU921075
Sicyoniidae Ortmann, 1898						
*Sicyonia laevigata* Stimpson, 1871	ULLZ7192	**JX403868**	**JX403850**	**JX403832**	**JX403811**	**JX403907**
*Sicyonia ingentis* (Burkenroad, 1938)	KC4279	**JX403880**	GQ487492	**JX403838**	N/A	GQ487518
Solenoceridae Wood-Mason, 1891						
*Hymenopenaeus debilis* Smith, 1882	ULLZ8531	**JX403866**	**JX403848**	**JX403830**	**JX403809**	**JX403890**
*Mesopenaeus tropicalis* (Bouvier, 1905)	ULLZ8364	**JX403867**	**JX403849**	**JX403831**	**JX403810**	**JX403891**
*Pleoticus robustus* (Smith, 1885)	ULLZ10956	**JX403881**	**JX403857**	**JX403839**	**JX403821**	**JX403901**
*Solenocera necopina* Burkenroad, 1939	ULLZ6705	**JX403871**	**JX403853**	**JX403835**	**JX403814**	**JX403894**
Sergestoidea Dana, 1852						
Sergestidae Dana, 1852						
*Sergia hansjacobi* Vereshchaka, 1994	ULLZ11552	**JX403883**	**JX403859**	**JX403841**	**JX403823**	**JX403903**
*Sergia* nr. *robusta*	ULLZ8089	**JX403878**	EU868710	EU868807	GQ487509	GQ487519
*Deosergestes corniculum* (Krøyer, 1855)	ULLZ11598	**JX403885**	**JX403861**	**JX403843**	**JX403825**	**JX403905**

**Figure 2:**
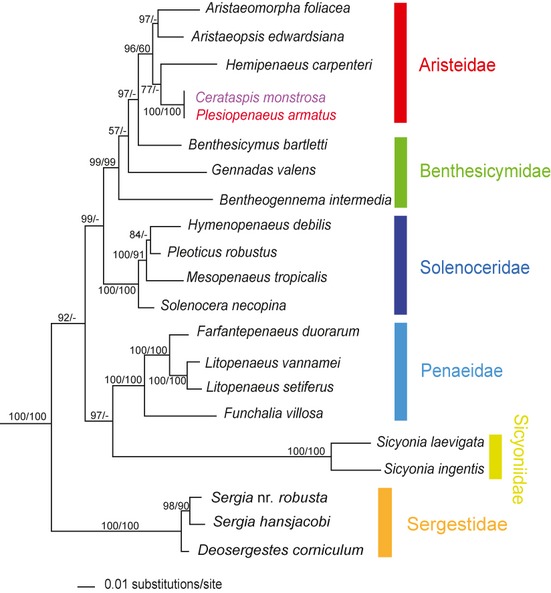
Bayesian (BAY) phylogram for selected dendrobranchiate taxa (*n* = 21) and outgroups (*n* = 6) based on a 12S (mtDNA), 16S (mtDNA), 18S (nDNA), 28S n(DNA) and H3 n(DNA) concatenated dataset. BAY posterior probabilities and ML bootstrap values are represented as percentages and noted above or below the branches (BAY/ML). Values <50% are not shown and represented by “-” Vertical colored bars indicate families within Decapoda. Outgroups not shown.

**Figure 3:**
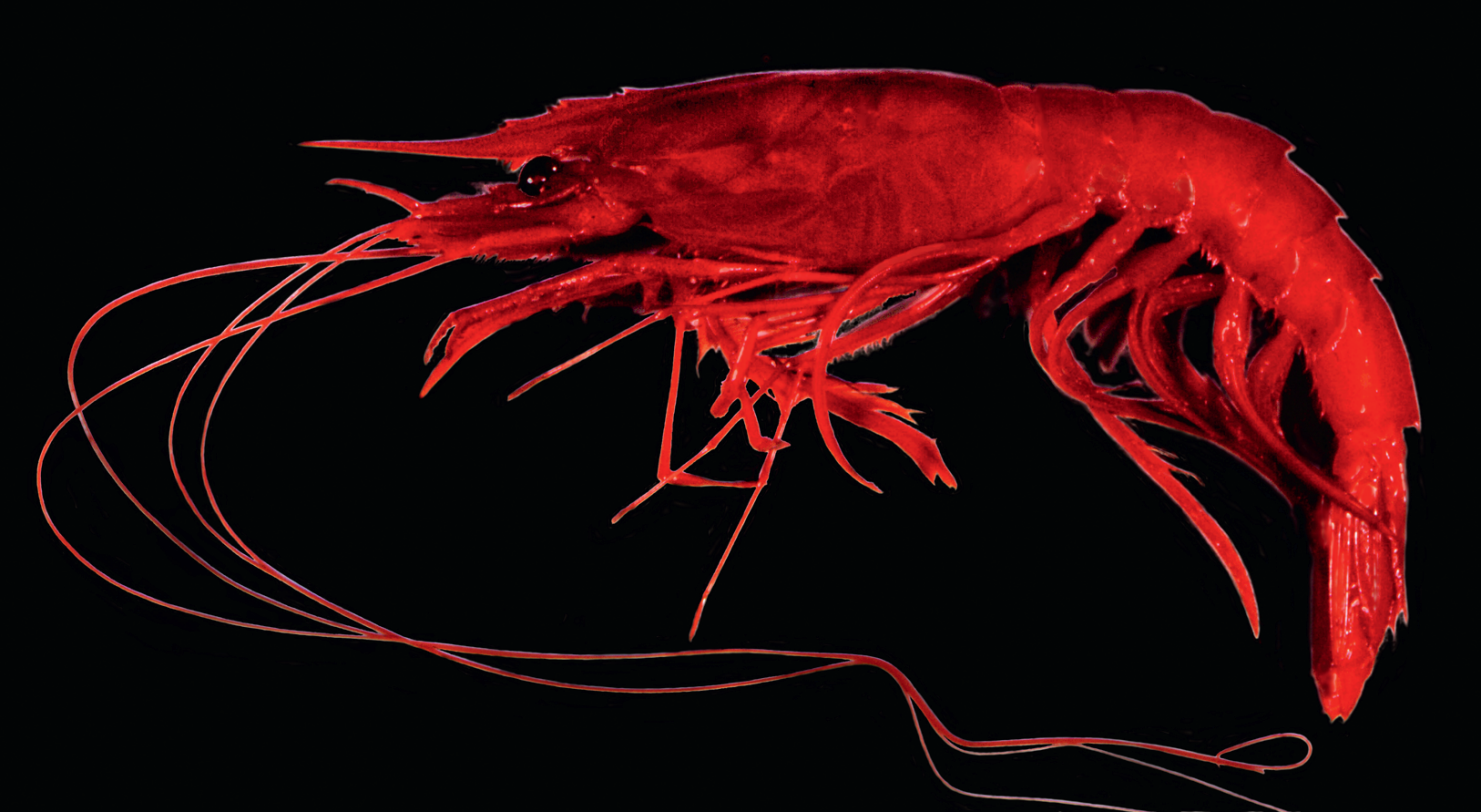
*Plesiopenaeus armatus* (median carapace length 136 mm), the inferred adult form of *Cerataspis monstrosa* as indicated by the 99.96% sequence identity across 5 genes. [Credit: W. Pequegnat, 1971, female from 3250 m, northwestern Gulf of Mexico]. The individual included in the analysis was collected on 8 June 2000 in the northern Gulf of Mexico from 3050 m at 27˚59.43′N, 86˚43.36′W by G. Rowe et al.

Phylogenetic analysis (Fig. 2) places *C. monstrosa* as identical to the deep-sea penaeoid shrimp *P. armatus* (Figs. 1,3). Moreover, our sequencing efforts of 4136 basepairs over five genes (12S, 16S, 18S, 28S, H3) resulted in a near perfect (99.96%) genetic match between these two “species.” Individual gene trees were not in conflict, with 12S and 16S resolving shallow branches and 28S, 18S, and H3 resolving middle to deep branches. All genetic markers in our analysis were carefully selected to include enough variation to detect species-level differences and resolve systematic placement. Historically, these nuclear and mitochondrial markers have demonstrated their utility in decapod taxonomic, systematic, and barcoding studies (Bracken et al. 2010; Grave et al. 2010; Puillandre et al. 2011). For each gene, the level of divergence between *P. armatus* and *C. monstrosa* is considerably less (∼0.049−0.18%) when compared with estimates among other congeneric decapod (∼2.2−10%, Toon et al. 2009) and aristeid (∼3%, pers. observation based on 16S GenBank data, JF899802, GU972651) species. We therefore conclude that *P. armatus* and *C. monstrosa*, respectively, represent adult and larval forms of the same species, and recommend both henceforth be referred to as *P. armatus* (see Taxonomy Note).

Larval–adult linkages allow for the advancement of understanding in ecology, systematics, and taxonomy, and in the case of *C. monstrosa*, both deep-sea and plankton biology. Linkages shed light on the distribution, ecology, and life history of a species. Known occurrences of *C. monstrosa* and adults of *P. armatus* overlap in geographic distribution, which further solidifies the larval–adult identification. Although the first report of *C. monstrosa* in the Gulf of Mexico was relatively recent (Franks and Russell 2008), the larval form appears to be circumglobally distributed in oceanic mid-water pelagic communities, near-surface plankton communities, or in association with surface rafts of *Sargassum* (Heegaard 1966a; Morgan et al. 1985). The reduced abdomen and armored thorax suggests that *C. monstrosa* has an extended pelagic life, as proposed in previous reports (Bouvier #b^101^). The adult counterpart, *P. armatus*, is of similar cosmopolitan distribution, albeit as a true abyssal species ranging widely in deep-ocean basins to depths of at least 5060 m (Gore 1985; Pérez Farfante and Kensley 1997). Specifically throughout the Gulf of Mexico, adults of *P. armatus* have been reported from depths of 1,764–3,600 m (Roberts and Pequegnat 1970; Crosnier and Forest 1973; Pérez Farfante and Kensley 1997; Felder and Camp 2009). Thus, linking of the adult to larval form provides novel insight into the life history of this species from a mid-water pelagic larva to an abyssal adult. This furthermore establishes the adult source population for larvae that are a common food of pelagic fishes. Findings from this study suggest a second known “species” of *Cerataspis*, *C. petiti*, is likely a larval stage of the only other known species of *Plesiopenaeus* (*P. coruscans*). Affinities of the closely related and equally bizarre “larval” species *Cerataspides longiremus*, first described as *Cerataspis* by Dohrn (1871) and placed in the genus *Cerataspides* by Bonnier (1899), may well be a larval stage of an unidentified member of the genus *Plesiopenaeus* or of another aristeid shrimp (Dohrn 1871; Bonnier 1899). Similar approaches, as applied here, can be used to confirm these larval–adult linkages once material of these rare individuals becomes available for molecular systematic studies.

Genetic techniques cross-validated with larval rearing protocols are the preferred method of identifying adult–larval linkages. However, molecular phylogenetic tools, as applied here, provide a powerful alternative to traditional approaches dependent on rearing of otherwise unidentifiable larvae. In this case, the combined application of modern DNA techniques with robust phylogenetic methodology allowed us to solve this 184-year-old mystery of the “monster larva” of the deep.

## Methods

### Taxon sampling

One specimen of *C. monstrosa* was collected on July 2 2009 in the northeastern Gulf of Mexico from a depth of 420 m at 27˚05.996′N, 86˚25.916′W during mid-water trawl collection by cruise participants aboard NOAA Ship Gordon Gunter. As past studies have suggested an affinity between *C. monstrosa* and penaeoids, but specifically the family Aristeidae, taxon sampling was focused within these lineages. Additional taxa from the Aristeidae (including species and/or specimens of *Plesiopenaeus*) were not included due to the difficulty in collecting deep-sea organisms, rarity in nature, and/or unavailability of molecular grade tissues. In total, 21 ingroup taxa across the dendrobranchiate superfamilies Penaeoidea and Sergestoidea were included in the phylogenetic analysis (Table 1). Representatives of other shrimp-like groups including carideans, euphausiaceans, and stenopodideans were included as outgroups (not shown).

### Sequencing and phylogenetic analyses

Total genomic DNA was extracted from either the abdominal muscle or gill using the Qiagen DNeasy® rBlood and Tissue Kit (Cat. No. 69582; Qiagen, California), QIAamp DNA Mini Kit (Qiagen) (Cat. No. 51304) or QIAamp DNA Micro Kit (Qiagen) (Cat. No. 56304). Two mitochondrial (12S, 16S) and three nuclear genes (18S, 28S, H3) were selected due to their range of phylogenetic utility and different inheritance patterns. Initially, we tried to amplify the barcoding region of COI (Folmer region), however, multiple attempts failed in our targeted species. Additionally, 16S, 12S, and partial 28S are often used in systematic and decapod barcoding studies and contain enough variation to detect species-level differences (Bracken et al. 2010; Grave et al. 2010; Puillandre et al. 2011). Genes were amplified using one or more sets of primers. These included the mitochondrial genes 16S large ribosomal subunit (∼550 bp, Crandall and Fitzpatrick 1996) and 12S small ribosomal subunit (∼400 bp, Buhay et al. 2007), in addition to the nuclear genes 28S large ribosomal subunit (∼2500 bp, Whiting et al. 1997; Whiting 2002; Palero et al. 2008) 18S small ribosomal subunit (∼1800 bps, Medlin et al. 1988; Whiting et al. 1997; Apakupakul et al. 1999; Whiting 2002; Bracken et al. 2009), and protein-coding histone 3 (H3) (∼350 bp, Colgan et al. 1998). Polymerase chain reaction (PCR) amplifications were preformed in 25–50 μL volumes followed by cycle sequencing reactions using an Applied Biosystems 9800 Fast Thermal Cycler (Applied Biosystems, Foster City, CA, USA). Forward and reverse sequencing products were run on an ABI 3730xl DNA Analyzer 96-capillary automated sequencer in the Brigham Young University (BYU) sequencing center.

After sequence cleaning and editing using Sequencher 4.8 (GeneCodes, Ann Arbor, MI, USA), all sequences were checked for contamination and/or pseudogenes by following suggestions by Song et al. 2008 and BLAST searches. Individual alignments were created using MAFFT (E-INS-I option), and GBlocks was used to omit highly divergent and poorly aligned positions. Individual gene trees were generated using Maximum Likelihood (ML, Felsenstein 1981) analyses to ensure similar topologies and gene histories. Alignments were concatenated into a single dataset consisting of 4136 basepairs.

A phylogenetic approach was selected over alternative species delimitation methods (Yang and Rannala 2010; Ence and Carstens 2011; Masters et al. 2011) due to the limited availability of material for inclusion in the analysis. However, in studies where multiple individuals per species are obtainable, we suggest using a combination of phylogenetic and species delimitation approaches. A ML analysis (Felsenstein 1981) was conducted using RAxML (Randomized A(x)ccelerated Maximum Likelihood) (Stamatakis et al. 2005) with computations performed on the computer cluster of the Cyberinfrastructure for Phylogenetic Research Project (CIPRES 2.0) at the San Diego Supercomputer Center. The model of evolution that best fit the individual datasets was determined using MODELTEST 3.7 (Posada and Crandall 1998). The Bayesian (BAY) analysis was conducted in MrBayes v3.1.2b4 (Huelsenbeck and Ronquist 2001) on the Marylou5 Computational Cluster at Brigham Young University. Three independent runs were performed (each consisting of 20 chains and 10 swaps). Each analysis ran for 20,000,000 iterations, which we thinned to every 1000th iteration. Bootstrap support values (1000 pseudoreplicates) (Felsenstein 1985) and posterior probabilities (documented as percentages) are presented on the BAY phylogram (Fig. 2).

## Taxonomy Note

The International Code of Zoological Nomenclature (http://iczn.org/) requires via its Principle of Priority (Article 23) that an older available name (in this case *C. monstrosa* Gray 1828) has precedence over a younger name (*P. armatus* (Bate 1881)) in a case where “two or more generations, forms, stages, or sexes of a species are named as different nominal taxa” (ICZN 23.3.2.2). If strictly applied in the current case, the two species known as *P. armatus* (Bate 1881) and *Plesiopenaeus coruscans* (Wood-Mason in Wood-Mason & Alcock, 1891) should henceforth be known as *Cerataspis armatus* (Bate 1881) and *Cerataspis coruscans* (Wood-Mason in Wood-Mason & Alcock 1891), respectively. However, the ICZN also has the plenary power to modify an application of the Code “if such application would in its judgment disturb stability or universality or cause confusion” (Article 81.1). As the genus name *Plesiopenaeus* Bate, 1881, is widely recognized and used to refer to the adults (e.g., in Perez-Farfante and Kensley 1997; Tavares and Martin 2010; Grave and Fransen 2011) as compared to the relatively infrequent use of *Cerataspis* (Gray 1828) (use of which has been restricted to larval forms, which are rare), we are applying to the ICZN to use its plenary action to suppress *Cerataspis* in favor of *Plesiopenaeus* for stability and to avoid confusion. If our application is accepted, the term “cerataspis” could continue to be used as an informal name for these distinctive larval forms, just as the names zoea, megalopa, glaucothoe, eryoneicus, and other names once thought to represent decapod adults are still employed.
